# Hidden blood loss after proximal femur fractures fixation: analysis of the first three postoperative days and influence of anticoagulants

**DOI:** 10.1007/s00402-026-06330-3

**Published:** 2026-05-16

**Authors:** Jorge Mayor, Marcel Winkelmann, Lea-Sophie Schwinn, Hür Özbek, Gökmen Aktas, Tarek Omar-Pacha, Swantje Oberthür, Rike-Herta Krammig, Axel Gänsslen, Stephan Sehmisch, Jan-Dierk Clausen

**Affiliations:** Department of Orthopedic Trauma, Hannover Medical School, Hanover, Germany

**Keywords:** Hidden blood loss, Anticoagulants, Proximal femur fracture, Elderly, Blood management, Perioperative risk

## Abstract

**Purpose:**

Hidden blood loss (HBL) is an underrecognized contributor to total perioperative blood loss after hip fracture surgery. This study aimed to quantify HBL during the first three postoperative days following proximal femur fracture fixation and to assess the impact of preoperative anticoagulant therapy on blood loss and transfusion requirements.

**Methods:**

A retrospective cohort study was conducted including patients aged ≥ 65 years who underwent surgical fixation of proximal femur fractures at a level I trauma center between January 2020 and December 2022. Total blood loss was estimated from perioperative hemoglobin changes and calculated blood volume. Patients were stratified according to anticoagulant use, and logistic regression analysis was performed to identify independent predictors of increased blood loss.

**Results:**

Among 260 patients analyzed, median total blood loss was 1184 ml, and 25% experienced losses exceeding 1675 ml. High blood loss correlated significantly with anticoagulant use, higher BMI, and longer operative time (*p* < 0.001). In multivariate analysis, anticoagulant therapy (particularly direct oral anticoagulants) remained an independent predictor of high blood loss (OR 3.06; 95% CI 1.47–6.37; *p* = 0.003). Patients in the highest quartile required significantly more transfusions, though short-term mortality did not differ.

**Conclusion:**

Hidden blood loss after proximal femur fracture surgery is frequent and clinically relevant. Anticoagulant use, elevated BMI, and prolonged operative duration independently increase bleeding risk. Awareness of these predictors may support optimized perioperative management and transfusion planning in elderly fracture patients.

**Supplementary Information:**

The online version contains supplementary material available at 10.1007/s00402-026-06330-3.

## Introduction

Proximal femur fractures are among the most common injuries in elderly individuals and represent a growing challenge for healthcare systems in industrialized nations. Due to demographic aging and increasing life expectancy, the incidence of such fractures is projected to rise substantially—from an estimated 1.26 million in 1990 to 2.6 million by 2025 and potentially 4.5 million by 2050 worldwide [1]. Surgical treatment of these fractures aims to restore patient mobility and independence, yet perioperative and postoperative complications remain common and significantly affect outcomes.

One critical factor is blood loss, which includes both visible intraoperative bleeding and hidden blood loss (HBL). While intraoperative blood loss is usually well quantified, HBL often goes unrecognized and may lead to significant postoperative anemia. HBL is defined as the discrepancy between measured intraoperative blood loss and the actual decline in hemoglobin over the perioperative period, accounting for blood sequestration in tissues, hemolysis, and undetected bleeding [2, 3].

Several studies have highlighted the magnitude and clinical relevance of HBL. Sehat et al. analyzed 202 patients undergoing total hip and knee arthroplasty and found that hidden blood loss often exceeded visible blood loss, particularly after knee procedures [2]. Foss et al. extended these findings to hip fracture surgeries, using the formula developed by Nadler, Hidalgo, and Bloch to estimate blood volume and calculate total loss [4, 5]. These methods have since become standard in quantifying occult blood loss in orthopedic surgery. Research has also focused on the effect of pharmacological interventions, such as tranexamic acid, in reducing HBL. Good et al. and Oliva-Moya et al. both demonstrated that perioperative administration of tranexamic acid significantly reduces both external and hidden bleeding after joint replacement surgery [6, 7].

Despite this growing body of literature, the role of anticoagulant therapy in perioperative blood loss—especially HBL—remains poorly understood in proximal femur fractures. Given that many elderly patients receive anticoagulation for the prevention of thromboembolic events, understanding its impact on perioperative bleeding is critical for surgical planning and transfusion management [8].

Although prior studies have documented substantial blood loss in orthopedic trauma and arthroplasty, there remains limited evidence on how modern anticoagulants influence hidden blood loss specifically in this high-risk population. Given the increasing prevalence of anticoagulant use among geriatric patients, our aim was to fill this gap by identifying clinical predictors of elevated HBL, thereby supporting more individualized perioperative management.

## Patients and methods

This retrospective cohort study analyzed data from 293 patients with surgically treated proximal femur fractures at a level I trauma center between October 1, 2020, and December 31, 2022. 260 patients matched the inclusion criteria. The study aimed to investigate the influence of anticoagulant therapy on perioperative blood loss—particularly hidden blood loss (HBL)—during the first three postoperative days.

Patients were eligible for inclusion if they met the following criteria:

- Age ≥ 65 years.
- Isolated proximal femur fracture (pertrochanteric or femoral neck).
- Primary surgical treatment with either proximal femoral nailing, total hip arthroplasty or dual mobility hemiarthroplasty, based on fracture morphology.
- Complete postoperative follow-up of at least 3 days.

Exclusion criteria included:

- Age < 65 years.
- Follow-up < 3 days.
- Incomplete preoperative laboratory results.
- Incomplete preoperative medical history regarding anticoagulants.
- Pathologic fractures, periprosthetic fractures.

## Study groups

Patients were stratified into two groups based on the presence or absence of oral anticoagulant therapy at admission (e.g., direct oral anticoagulants or vitamin K antagonists).

## Blood loss estimation

Perioperative blood loss was calculated based on the method by Gross [3], refined by Sehat et al. [2], and using estimated blood volume (EBV) as per the Nadler formula [5]:

1. Estimated Blood Volume (EBV):
  Women: EBV (L) = 0.356 × height (m)³ + 0.033 × weight (kg) + 0.183.
  Men: EBV (L) = 0.367 × height (m)³ + 0.032 × weight (kg) + 0.604.
2. Hemoglobin Loss (g):
  $$ \begin{aligned} {\mathrm{Hb}}\_{\mathrm{loss}} & ={\mathrm{EBV}} \times \left( {{\mathrm{Hb}}\_{\mathrm{admission}} -{\mathrm{Hb}}\_{\mathrm{day3}}} \right)\\ & \quad +{\text{transfused Hb mass}} \end{aligned}$$
3. Estimated Total Blood Loss (ml):
  $${\text{Total blood loss}}=\left( {{\mathrm{Hb}}\_{\text{loss }}/{\text{ Hb}}\_{\mathrm{admission}}} \right){\text{ }} \times {\text{ 1}}000$$

Here, transfused Hb mass was estimated at ~ 52 g per red cell unit [9].

### Statistical analysis

Data analysis was performed using IBM SPSS Statistics Version 24 (IBM Corp., Armonk, NY, USA).

- Continuous variables were expressed as mean ± standard deviation (SD) and compared using the Mann–Whitney U test.
- Categorical variables were reported as counts and percentages and compared using the Pearson chi-square test.

To assess associations between anticoagulant use and perioperative blood loss, univariate analysis was conducted first. Significant variables were then included in multivariate logistic regression models to identify independent predictors. The models adjusted for age, sex, BMI, fracture type, surgical method, and anticoagulant therapy. Odds ratios (OR) with 95% confidence intervals (CI) were reported, with statistical significance set at *p* < 0.05.

### Ethical approval

This study was conducted in accordance with the ethical principles outlined in the Declaration of Helsinki. Ethical approval was obtained from the ethics committee of the Hannover Medical School (No: 11274_BO_K_2024).

## Results

A total of 260 patients with surgically treated proximal femur fractures were included. The mean age was 81.2 ± 11.0 years, and 64.2% were female. The mean BMI was 24.3 ± 4.4 kg/m². Most fractures were pertrochanteric (61.5%) and treated predominantly with proximal femoral nailing systems (39.6%) or hemiarthroplasty (29.2%). Anticoagulant therapy was present in 29.2% of patients, with apixaban, coumarins, and rivaroxaban being the most commonly used agents. The average operative time was 94.8 ± 47.6 min. A summary of baseline characteristics is provided in Table 1.

**Table 1:** Baseline demographic and clinical characteristics of the study population (n = 260) stratified by blood loss (increased vs decreased). Continuous variables are presented as mean ± standard deviation (SD), and categorical variables as number (percentage). Patients with increased blood loss were significantly younger, taller, heavier, and had higher BMI compared to those with decreased blood loss, while no significant differences were observed for sex, fracture type, surgical technique, ASA score, length of hospital stay, or mortality.

Variable	Total [*n* = 260]	Blood loss ↑ [*n* = 130]	Blood loss ↓ [*n* = 130]	*p* value
Sex (male); n (%)	95 (36.5)	55 (42.3)	40 (30.8)	0.0531
Age [years]; Mean ± SD	81.0 ± 11.0	79.3 ± 11.7	82.7 ± 10.0	0.0431
Height [cm]; Mean ± SD	167 ± 10	169 ± 10	166 ± 9	0.0052
Weight [kg]; Mean ± SD	68.6 ± 14.6	73.1 ± 15.2	64.0 ± 12.6	< 0.0012
BMI [kg/m²]; Mean ± SD	24.4 ± 4.4	25.4 ± 4.5	23.3 ± 4.0	< 0.0012
Fracture: Intracapsular; n (%)	120 (46.2)	59 (45.4)	61 (46.9)	0.8041
Extracapsular; n (%)	140 (53.8)	71 (54.6)	69 (53.1)	0.8041
*Surgical technique; n (%)*				
HA	76 (29.2)	37 (28.5)	39 (30.0)	0.8641
DHS	62 (23.8)	31 (23.8)	31 (23.8)	0.8641
THA	18 (6.9)	10 (7.7)	8 (6.2)	0.8641
PFN	103 (39.6)	51 (39.2)	52 (40.0)	0.8641
Screw osteosynthesis	1 (0.4)	1 (0.8)	0 (0)	0.8641
*ASA score; n (%)*				
I	7 (2.7)	1 (0.8)	6 (4.6)	0.0891
II	99 (38.1)	44 (33.8)	55 (4.2)	0.0891
III	135 (51.9)	74 (56.9)	61 (46.9)	0.0891
IV	19 (7.3)	11 (8.5)	8 (6.2)	0.0891
Hospital stay [days]; Mean ± SD	8.5 ± 5.0	8.8 ± 5.5	8.2 ± 4.4	0.3672
Mortality; n (%)	16 (6.2)	10 (7.7)	6 (4.6)	0.3021

Patients were stratified into two groups based on the median blood loss: ≥1184 ml (high loss group) and < 1184 ml (low loss group). Patients in the high blood loss group were significantly younger (79.3 ± 11.7 vs. 82.7 ± 10.0 years, *p* = 0.043), taller (169 ± 10 vs. 166 ± 9 cm, *p* = 0.005), heavier (73.1 ± 15.2 vs. 64.0 ± 12.6 kg, *p* < 0.001), and had a higher BMI (25.4 ± 4.5 vs. 23.3 ± 4.0 kg/m², *p* < 0.001). No differences were observed in sex distribution, ASA score, or fracture classification.

Postoperative day 3 hemoglobin was significantly lower in the high blood loss group (8.3 ± 1.2 vs. 10.0 ± 1.4 g/dl, *p* < 0.001). Operative duration was also significantly longer (106.8 ± 49.8 vs. 82.7 ± 44.1 min, *p* < 0.001). Anticoagulant use was more common in the high blood loss group (37.5% vs. 20.4%, *p* = 0.006). Supplementary Table S1 presents detailed group comparisons.

Patients in the highest quartile of blood loss (> 1675 ml) were analyzed separately. This group showed a higher prevalence of anticoagulant therapy, especially DOACs and coumarins. Surgical procedures in this subgroup included a higher proportion of total hip arthroplasty and proximal femoral nailing, which were associated with greater blood loss.

These patients required more frequent red blood cell transfusions (44.6% vs. 13.8%, *p* < 0.001) and received higher total hemoglobin mass (110.3 ± 97.8 vs. 55.6 ± 20.1 g, *p* < 0.001). Transfusion volumes ≥ 1000 ml occurred exclusively in this subgroup.

Supplementary Table S2 includes full data for this comparison.

## Logistic regression analysis

Two logistic regression models were performed to identify independent predictors of elevated perioperative blood loss. In Model 1 (blood loss ≥ 1184 ml), BMI was an independent predictor (OR 1.12 per kg/m²; 95% CI 1.05–1.20; *p* < 0.001). Age showed a mild protective effect (OR 0.97 per year; 95% CI 0.95–1.00; *p* = 0.037). Anticoagulant use showed a trend (OR 1.81; 95% CI 0.88–3.69; *p* = 0.105), though not statistically significant. Nagelkerke R² = 0.129. In Model 2 (blood loss ≥ 1675 ml), anticoagulant use was a significant predictor (OR 3.06; 95% CI 1.47–6.37; *p* = 0.003). BMI remained significant (OR 1.08 per kg/m²; 95% CI 1.01–1.16; *p* = 0.026), and male sex showed a borderline association (OR 1.85; 95% CI 1.00–3.43; *p* = 0.051). Coagulation parameters (PTT, INR) did not correlate with blood loss. Nagelkerke R² = 0.126. Despite higher transfusion requirements in high blood loss groups, no significant differences were found in short-term outcomes such as in-hospital mortality (7.7% vs. 4.6%, *p* = 0.302) or discharge disposition (*p* = 0.795).

## Discussion

This retrospective cohort study investigated hidden blood loss (HBL) during the early postoperative period following proximal femur fracture fixation in a geriatric population, with a specific focus on the influence of anticoagulant therapy. Our findings suggest that HBL is a significant and often underestimated component in these patients. Key patient- and treatment-related factors—most notably anticoagulant use, higher BMI, and prolonged operative duration—were associated with increased HBL.

The observed decline in hemoglobin levels despite controlled intraoperative conditions reinforces the clinical relevance of occult blood loss. Nearly 50% of patients in our cohort experienced total blood losses exceeding 1000 ml, with 25% surpassing 1675 ml. These findings are consistent with previous research by Sehat et al. and Gross et al., who demonstrated that conventional intraoperative measurements underestimate total blood loss in orthopedic procedures [2, 3].

Our data highlight the significant role of anticoagulant therapy in increasing blood loss, particularly among patients receiving direct oral anticoagulants (DOACs). This finding corroborates recent reports showing that DOACs are associated with higher transfusion rates and prolonged postoperative anemia in fracture surgery, even when INR/PTT levels appear normal [8, 10]. The pharmacodynamic variability of DOACs, combined with the absence of universally available reversal agents, complicates perioperative management and introduces additional bleeding risk in the acute setting [11].

Furthermore, BMI emerged as an independent predictor of greater HBL, echoing recent studies that link obesity to increased intra- and postoperative bleeding due to expanded soft tissue compartments and greater surgical complexity [12]. Prolonged operative duration similarly correlated with elevated HBL, likely reflecting increased tissue trauma and exposure time. This is in line with the findings of Ross et al., who reported that for every 15-minute increase in operative time, the odds of requiring a transfusion increased by 35% [13].

From a procedural standpoint, intramedullary nailing (PFN) was associated with higher total and hidden blood loss compared to dynamic hip screw (DHS) fixation or hemiarthroplasty. This is consistent with earlier studies demonstrating that canal reaming and intramedullary pressure elevation in PFN contribute to occult bleeding [14]. While hemiarthroplasty may involve more intraoperative blood loss, its overall hidden loss appears lower in comparison, especially when cemented techniques are used [9].

Despite increased transfusion requirements, we did not observe significant differences in short-term mortality or discharge status. This may reflect the effectiveness of modern transfusion thresholds and perioperative care. However, HBL should not be dismissed as clinically irrelevant: recent evidence links perioperative anemia and occult bleeding to delayed mobilization, increased length of hospital stay, and impaired rehabilitation, especially in frail older adults [15, 16].

From a clinical perspective, our findings suggest that preoperative anticoagulant status, BMI, and expected operative time should be factored into risk stratification and blood management planning. This could include more aggressive early monitoring of hemoglobin, individualized transfusion triggers, and consideration of reversal strategies in selected DOAC patients. These adjustments may help mitigate the impact of HBL and reduce downstream complications [17, 18].

This study is limited by its retrospective design and reliance on formula-based blood loss estimation, which may introduce variability. The relatively small subgroup sizes for individual anticoagulants limit direct comparisons. Long-term functional outcomes were not assessed, and this represents an important area for future investigations.

Hidden blood loss represents a clinically relevant and underrecognized phenomenon following proximal femur fracture fixation in elderly patients. Anticoagulant use, elevated BMI, and longer operative duration are independently associated with increased HBL. These findings support the integration of risk-based perioperative strategies to optimize outcomes and aim to improve resource planning.

## Limitations

This study is based on a retrospective design and is therefore subject to the inherent methodological limitations associated with such analyses. Furthermore, factor Xa levels were not determined in all patients, which precluded a clear differentiation between anticoagulation with vitamin K antagonists (coumarins) and direct oral anticoagulants (DOACs). For this reason, the assessment of these parameters has recently been implemented as part of the geriatric trauma assessment in our institution. Further prospective studies are required to allow for a more precise differentiation in the future.

## Conclusion

Hidden blood loss (HBL) is a clinically significant yet often underestimated factor in the surgical management of proximal femur fractures especially in elderly patients. Our study suggests that HBL frequently exceeds intraoperative estimates and contributes substantially to total perioperative blood loss. Notably, patients receiving anticoagulant therapy—particularly DOACs—are at elevated risk, as are those with higher BMI and longer operative times. These factors appear to act independently and cumulatively, emphasizing the multifactorial nature of occult blood loss.

Despite no immediate impact on short-term mortality, the extent of hidden blood loss may influence transfusion needs, early functional recovery, and rehabilitation outcomes. Given the growing demographic of elderly, comorbid patients undergoing hip fracture surgery, heightened awareness and targeted perioperative strategies are warranted. These may include early risk stratification, careful selection of surgical approach, individualized transfusion thresholds, and where appropriate, the reversal or temporary modification of anticoagulant therapy.

Further prospective, multicenter studies are needed to validate predictive models of HBL and assess its impact on long-term outcomes such as functional independence, mobility, and quality of life.

## Supplementary Information

Below is the link to the electronic supplementary material.

Supplementary file1

## Data Availability

No datasets were generated or analysed during the current study.
