# Supplementary material for: Hidden blood loss after proximal femur fractures fixation: analysis of the first three postoperative days and influence of anticoagulants
Source: Arch Orthop Trauma Surg. 2026 May 16;146(1):188. doi: 10.1007/s00402-026-06330-3 (PMC13179911; doi:10.1007/s00402-026-06330-3)
Supplement: Supplementary file 1 — Supplementary file1 [file 402_2026_6330_MOESM1_ESM.docx]

# Supplementary Tables

## Supplementary Table S1. Comparison between patients with high vs. low total blood loss (≥1184 ml vs <1184 ml)

| **Variable** | **High Blood Loss (≥1184 ml)** | **Low Blood Loss (<1184 ml)** | **p-value** |
| --- | --- | --- | --- |
| **Age (years)** | 79.3 ± 11.7 | 82.7 ± 10.0 | 0.043 |
| **Height (cm)** | 169 ± 10 | 166 ± 9 | 0.005 |
| **Weight (kg)** | 73.1 ± 15.2 | 64.0 ± 12.6 | <0.001 |
| **BMI (kg/m²)** | 25.4 ± 4.5 | 23.3 ± 4.0 | <0.001 |
| **Post-op Hb (g/dl)** | 8.3 ± 1.2 | 10.0 ± 1.4 | <0.001 |
| **Operative time (min)** | 106.8 ± 49.8 | 82.7 ± 44.1 | <0.001 |
| **Anticoagulant use (%)** | 37.5% | 20.4% | 0.006 |
| **Transfusion needed (%)** | 44.6% | 13.8% | <0.001 |
| **Transfused Hb (g)** | 110.3 ± 97.8 | 55.6 ± 20.1 | <0.001 |

Continuous variables are presented as mean ± SD; categorical variables as n (%). Mann–Whitney U test or Pearson Chi² test were applied as appropriate.

## Supplementary Table S2. Comparison between patients in the highest quartile (>1675 ml) and the remaining patients

| **Variable** | **Upper Quartile (>1675 ml)** | **Other Patients** | **p-value** |
| --- | --- | --- | --- |
| **Sex (male)** | 31 (48.4%) | 64 (32.7%) | 0.023 |
| **Age (years)** | 79.4 ± 11.5 | 81.5 ± 10.8 | 0.192 |
| **BMI (kg/m²)** | 25.6 ± 3.9 | 24.0 ± 4.5 | 0.002 |
| **Operative time (min)** | 106.8 ± 49.8 | 82.7 ± 44.1 | <0.001 |
| **Anticoagulant use (%)** | 37.5% | 20.4% | 0.006 |
| **PTT (s)** | 28.6 ± 4.5 | 28.4 ± 6.4 | 0.114 |
| **INR** | 1.05 ± 0.29 | 1.07 ± 0.43 | 0.080 |
| **Transfusion needed (%)** | 44.6% | 13.8% | <0.001 |

Continuous variables are presented as mean ± SD; categorical variables as n (%). Mann–Whitney U test or Pearson Chi² test were applied as appropriate.
